# Health Systems Responsiveness in Addressing Indigenous Residents' Health and Mental Health Needs Following the 2016 Horse River Wildfire in Northern Alberta, Canada: Perspectives From Health Service Providers

**DOI:** 10.3389/fpubh.2021.723613

**Published:** 2021-12-02

**Authors:** Kayla M. Fitzpatrick, T. Cameron Wild, Caillie Pritchard, Tara Azimi, Tara McGee, Jodi Sperber, Lorraine Albert, Stephanie Montesanti

**Affiliations:** ^1^School of Public Health, University of Alberta, Edmonton, AB, Canada; ^2^Department of Earth and Atmospheric Sciences, University of Alberta, Edmonton, AB, Canada; ^3^Kee Tas Kee Now Tribal Council, Atikameg, AB, Canada; ^4^Mikisew Cree First Nation, Fort Chipewyan, AB, Canada; ^5^Centre for Healthy Communities, School of Public Health, University of Alberta, Edmonton, AB, Canada

**Keywords:** wildfire, health systems responsiveness, service provision, Indigenous health, disaster recovery, psychosocial supports, mental health, health equity

## Abstract

Following the 2016 Horse River Wildfire in northern Alberta, the provincial health authority, the ministry of health, non-profit and charitable organizations, and regional community-based service agencies mobilized to address the growing health and mental health concerns among Indigenous residents and communities through the provision of services and supports. Among the communities and residents that experienced significant devastation and loss were First Nation and Métis residents in the region. Provincial and local funding was allocated to new recovery positions and to support pre-existing health and social programs. The objective of this research was to qualitatively describe the health systems response to the health impacts following the wildfire from the perspective of service providers who were directly responsible for delivering or organizing health and mental wellness services and supports to Indigenous residents. Semi-structured qualitative interviews were conducted with 15 Indigenous and 10 non-Indigenous service providers from the Regional Municipality of Wood Buffalo (RMWB). Interviews were transcribed verbatim and a constant comparative analysis method was used to identify themes. Following service provider interviews, a supplemental document review was completed to provide background and context for the qualitative findings from interviews. The document review allowed for a better understanding of the health systems response at a systems level following the wildfire. Triangulation of semi-structured interviews and organization report documents confirmed our findings. The conceptual framework by Mirzoev and Kane for understanding health systems responsiveness guided our data interpretation. Our findings were divided into three themes (1) service provision in response to Indigenous mental health concerns (2) gaps in Indigenous health-related services post-wildfire and (3) adopting a health equity lens in post-disaster recovery. The knowledge gained from this research can help inform future emergency management and assist policy and decision makers with culturally safe and responsive recovery planning. Future recovery and response efforts should consider identifying and addressing underlying health, mental health, and emotional concerns in order to be more effective in assisting with healing for Indigenous communities following a public health emergency such as a wildfire disaster.

## Introduction

A public health emergency such as, a natural disaster, places substantial burdens on the affected population's health and well-being, and on the health system's capacity to respond to changes in health and mental health needs. Emergency response plans and response activities are frequently designed to address and mitigate the immediate impact of the disaster on the health and safety of affected communities; however few plans fully anticipate and prepare for long-term health-related effects resulting from disasters (1). Natural disasters can also cause or exacerbate health-related concerns and inequities, overwhelming health service providers by dramatically increasing demand for their services after the disaster (2). The extent to which a disaster disrupts a health system's ability to care for population groups who are at a greater risk of poor health or mental health is an understudied but critical aspect of health system responsiveness after a disaster. Moreover, a health system overburdened by existing health disparities before a disaster may be the least well-equipped to respond to disaster compounded health and/or mental health concerns (1). For example, prior to Hurricane Katrina, one quarter of the New Orleans population lived below the poverty line. The devastating and long-lasting impacts of the hurricane resulted in an overwhelming load on an already overburdened health system (3). This paper focuses on services for Indigenous residents who were among those who experienced significant health and mental health impacts after the 2016 Horse River wildfire in northern Alberta, Canada. Qualitative research led by Montesanti et al. (4) documented heightened physical and emotional stress among First Nations and Métis Indigenous residents following this wildfire, as well as challenges that residents faced when accessing services and supports for health and mental health concerns. These findings are consistent with previous research showing that Indigenous communities are more vulnerable to the effects of wildfires (5, 6) compared to other sub-populations. Indigenous communities are often located in hazard-prone areas and inequitable access to services in addition to historical trauma can exacerbate vulnerability during a public health emergency (7–9). Further compounding this, a survey of adolescents aged 11–19 in the city of Fort McMurray showed worsening mental health 3.5 years following the Horse River wildfire, demonstrating the need for longer-term post-disaster support (10).

Responsive health systems during a public health emergency anticipate and adapt to changing needs, prepare for heightened health concerns, and promote access to effective, high-quality health services (11). Health system responsiveness was first conceptualized by the World Health Organization (WHO) in the 2000 World Health Report (12). That report defined the concept as follows: “…when institutions and institutional relationships are designed in such a way that they are cognisant and respond appropriately to the universally legitimate expectations of individuals…[including] safeguarding of rights of patients to adequate and timely care” (p. 3). This paper reports on results of a qualitative study to understand how the health system in the Regional Municipality of Wood Buffalo (RMWB) responded to the negative health and mental health effects experienced by Indigenous residents and communities during and following the 2016 Horse River wildfire.

### The Meaning of Health System Responsiveness for Indigenous Peoples

The concept of “health system responsiveness” has been used to understand peoples experience with the health system and the expectations that both health service users and health system actors have regarding how individuals should be treated and cared for when accessing health services (11). A person's interaction with their health system shapes their initial expectations and experiences of care (11, 13). Today, many aspects of health system responsiveness are aligned with key health system performance goals such as, quality care, safety, accessibility, appropriateness, and being patient-centered.

There are several key frameworks for understanding health systems responsiveness that focus on different aspects of responsiveness. The most widely used framework was proposed by the WHO in the early 2000s (12) which addresses an individual's experience within the health system along seven elements: dignity, autonomy, confidentiality, prompt attention, quality of amenities, access to social support networks, and choice of service provider. Building on the WHO framework, Mirzoev and Kane (11) proposed a conceptual framework that locates people's experiences when interacting with their health system at the center of health system responsiveness. For Indigenous patients, pervasive negative healthcare experiences and provider–patient relationships can prevent Indigenous peoples from accessing health services or avoid seeking care because of their perceived expectations of how they may be treated (14, 15). Beliefs, values, and assumptions held by health service providers shape their behaviors and interactions with patients and are influenced by discourses within society (16). In addition, the policy, legislative, historical, and social conditions that impact access for Indigenous peoples represent structural barriers to accessing health services (17, 18). Research shows that Indigenous peoples experience individual and systemic discrimination when seeking healthcare (19, 20), despite efforts within the health system to promote cultural responsiveness (20).

Valentine et al. (21) developed an alternative framework that outlines three key determinants of health system responsiveness. The first determinant–environment–defines the context of service provision, including health system expenditures, the structure of the health system, and available resources. The second determinant–agents defining need for care–refers to the role of users and providers in defining care needs and setting the context for care—for example, patient involvement in care decisions. The final determinant–the process of care and subsequent outcomes–is concerned with the process of seeking and receiving care at the micro (individual) level. The framework by Valentine et al. (21) provides a helpful approach for describing health system responsiveness for Indigenous peoples in Canada. Within the context of service provision, historical trauma, referring to the effects (direct and intergenerational) of colonization and residential schools on Indigenous peoples in Canada, contributes significantly to difficulties in accessing healthcare (22, 23) and has resulted in distrust of healthcare providers by the provincial and federal governments (24). The historical and ongoing forms of structural violence experienced by Indigenous peoples have unfolded against the broader context of neoliberal economic reforms, resulting in significant inequities in health. Health services are not typically designed to take into account the experiences of Indigenous peoples (25). For example, despite extensive evidence linking trauma and violence to multiple health problems (26, 27), these dynamics are rarely considered in the design and delivery of health services for Indigenous peoples. Thus, aligning health services with the needs of Indigenous peoples is lacking. Lastly, with respect to seeking and receiving care at the individual level, power dynamics and imbalances are particularly noteworthy and are at the root of inequities in healthcare. Previous negative experiences with healthcare services and/or healthcare providers among Indigenous peoples in Canada have repeatedly been found to create a barrier to accessing healthcare (28, 29). Thus, negative interactions with the health system shape Indigenous peoples' expectations of how they will be treated (30). Racism, discrimination, and harassment impede the development of trusting relationships with healthcare providers (24).

### Study Area and Context

The RMWB is home to five First Nation communities, including Mikisew Cree First Nation, Athabasca Chipewyan First Nation, Fort McKay First Nation, Fort McMurray First Nation, and Chipewyan Prairie Dene First Nation. This region is also home to many urban First Nations people as well as five Métis local organizations located in urban and rural Métis communities across the region. In 2016, the Canadian census (31) reported 71,480 residents with 6,565 identifying as Aboriginal in the RMWB. The estimated number of residents that were evacuated following the wildfire was 88,000 people; this number differs from the census as the RMWB has a large transient population as the local oil sands provides jobs for Canadians across the country and many workers do not permanently reside there. Prior to the large-scale development of the oil sands from the 1960s, Indigenous peoples, both First Nations and Métis, were the principal occupants of the region. Fort McMurray in the early 1960s, was a small and primarily Indigenous town, both demographically and culturally, in which traditional Indigenous ways of life and livelihoods, including hunting, fishing, gathering, and trapping, were combined with seasonal labor on the docks and the rail (32–34).

The Horse River wildfire started on May 1st, 2016 and ended on August 2nd, 2016. As mentioned above, approximately 88,000 people were forced to evacuate from RMWB on May 3rd, until approximately June 1st, 2016, and over 2,400 buildings and homes were destroyed. Like other residents of the RMWB, many Indigenous people and communities were also evacuated during the wildfire due to the threat of the fire or smoke. Residents spent several weeks away from their homes, jobs, schools, and communities. However, when it was time to return, many of Fort McMurray's urban Indigenous residents did not have houses to return to. Notably, the neighborhoods that were hit hard by the wildfire such as Abasand and Waterways have been anecdotally reported by the Métis Local in urban Fort McMurray to include a higher proportion of Indigenous residents compared to other neighborhoods in the city. Outside the Fort McMurray urban center, nearby Indigenous communities were also affected by the wildfire with some having to evacuate while others housed thousands of evacuees who sought refuge during the initial evacuation. For Indigenous peoples and communities that did not evacuate during the wildfire, the almost complete “shut down” of Fort McMurray made accessing food, medical services, employment, and other resources difficult for the duration of the evacuation.

This study described how health systems in Alberta responded to the short and long-term health and mental health effects experienced by Indigenous residents and communities during and following the 2016 Horse River. It is important to note that we are not looking at a single health system responsible for addressing health and mental health needs, rather we use a “systems” lens that speaks directly to the responsibilities across several relevant health and social care service delivery organizations (e.g., non-profit and charitable organizations, local community health centers, primary health care clinics, mental health, and addiction centers). Thus, we define health systems in our research to include a wide-range of local, provincial and federal health service-delivery organizations that include emergency and acute health services but go beyond them (i.e., curative and rehabilitative care), as well as organizations across sectors with mandates for health, wellness, and recovery. Our study addresses the following research questions: (1) How did the health and social care systems in Alberta respond to the health and mental health needs of Indigenous residents and communities post-wildfire? (2) How did health services providers adapt to the immediate health threats posed on Indigenous communities and residents in the RMWB?

## Methods

### Recruitment and Participants

Participants were recruited from the RMWB using a maximum variation sampling strategy (35). This sampling strategy was used to guide the selection and recruitment of service providers from different organizations with diverse perspectives and experiences. This included participant representation across rural and urban settings, First Nation (Cree and Dene individuals) and Métis population groups, and different service provider roles (e.g., nurse, social worker, health director). Participants were recruited through community engagement and relationship-building by connecting with local health directors and providers, and existing relationships and networks from community partners and recommendations from our Community Advisory Committee. Participants included a wide range of service providers that we have categorized as frontline workers, community and social workers, health directors and service team leads, and service delivery coordinators (Table 1). Service providers were invited to participate in an interview if they had directly provided or coordinated health or social services and supports during or following the 2016 wildfire for Indigenous residents or communities in the region.

**Table 1:** Breakdown of service provider categories.

**Service provider category**	***n***
Front line provider (e.g., physician, mental health counsellor)	4
Community and social work	10
Directors and service team leads (e.g., clinical director)	5
Coordinators (e.g., cultural coordinator)	6

### Study Procedure

The interview question guide was developed with the Community Advisory Committee, which included representation from health service providers drawn from different organizations that serve the RMWB Indigenous populations (e.g., Canadian Red Cross, Alberta Health Services, Local Friendship Center) as well as community members and Elder representation from the different Indigenous groups in the region (Métis, Dene, Cree). The active participation of the Community Advisory Committee began at the start of the project until completion, a process which empowers local communities by valuing and legitimizing their knowledge and by balancing power relationships among community and researchers for the project (36). With the research questions in mind, interview questions were developed from conversations with the community advisory committee and aimed to investigate impacts and recovery as it relates to the health and wellness of Indigenous groups, the mental health impacts and mental health services provided to Indigenous peoples and communities post-wildfire, culturally appropriate care and recommendations for service delivery (see Supplementary Material 1).

Semi-structured interviews were carried out with health and social care providers in the region to capture their perspectives on how the health needs and expectations of Indigenous peoples for accessing necessary services and supports during the traumatic experience of the wildfire, as well as their own expectations as providers in delivering support to the Indigenous population. Interviews were conducted with 15 Indigenous and 10 non-Indigenous service providers from the RMWB. As RMWB is a small region, no other demographic information was recorded other than their job title in order to maintain participant confidentiality. Interviews were ~1 h in duration and were completed by telephone or in person by the principal investigator (SM) or a trained community research assistant. Researchers explained the study and what to expect to participants and made sure they were aware they could stop the interview at any time. Participants signed an easily understood informed consent. Interviews were recorded on a recorder and transferred to a secure drive for analysis.

Following service provider interviews, a supplemental document review was completed to provide background and context for the qualitative findings. In addition, the document review allowed for a better understanding of the health systems response at a systems level following the wildfire. Documents were identified by recommendations from community leadership, complemented by a Google search. The google search included the search terms “Wildfire” and (“Northern Alberta” or “Fort McMurray” or “Regional Municipality of Wood Buffalo” or “Fort Chipewyan” or “Janvier” or “Conklin” or “Fort McMurray First Nation” or “Fort McKay” or “McMurray Metis”). Documents were included if they were published in English and if they discussed any impacts of the Horse River Fire on any Indigenous groups. Key questions were identified for the document review following the interviews and included: (1) Who funded health or mental health services during and following the wildfire? (2) Was there funding for Indigenous specific initiatives or programming? (3) What was the decision-making process for how funding was allocated? (4) What was the duration of the funding? (5) Were sustainability plans implemented? (6) Does the document or source include health-related findings resulting from the service provision? (7) Does the report include lessons learned on service provision for Indigenous peoples? Two researchers (KF, CP) thoroughly reviewed all relevant documents and extracted information pertaining to the questions. In total, 10 documents were analyzed (Table 2). Table 2 is in the Supplementary Materials.

**Table 2:** Post-disaster document information.

**Document title**	**Author(s)**	**Publication date (Year, Month)**
2016 Alberta fires: 1 year donor update	Canadian Red Cross	2017
Athabasca Tribal Council's health and wellness report	Athabasca Tribal Council	N/A
Community partnerships Alberta wildfires 2016 (webpage)	Canadian Red Cross	N/A
Community partnerships table terms of reference	Canadian Red Cross	2016, September
Rebuilding resilient indigenous communities in the RMWB: final report	Clark, T.	2018, October
May 2016 Wood Buffalo wildfire: post-incident assessment report	KPMG International network	2017, May
Regional Municipality of Wood Buffalo: lessons learned and recommendations from the 2016 Horse River Wildfire	KPMG International network	2017, July
RMWB 2016 wildfire recovery plan	NOR-EX Engineering	N/A
CARE Wood Buffalo executive summary: 2017–2018 community outreach	Alberta Health Services, Regional Municipality of Wood Buffalo, YMCA Supports for Wellness	2019, April
Home again: recovery after the Wood Buffalo wildfire	Government of Alberta	2016, November

### Data Analysis

Participant interviews were transcribed verbatim and imported into the qualitative data analysis software program QSR NVivo 12 for analysis. Two members of the research team (KF, TA) conducted coding and thematic analysis and consulted with the study PI (SM) and Co-I (CW) at the end of coding. One author on this manuscript (LA) is an Indigenous knowledge keeper as well as a service provider and used the two-eyed seeing approach to review and confirm our themes. Barlett and team (2012) define two eyed seeing as “to see from one eye with the strengths of Indigenous ways of knowing, and to see from the other eye with the strengths of Western ways of knowing, and to use both of these eyes together” [(37), p. 335]. The PI (SM) reviewed the coding for consistency and the PI and primary coder (KF) worked to identify, review, and name the themes. We used the framework method for the management and analysis of transcriptions. The framework method is a systematic method of categorizing and organizing data and is a flexible tool that is not aligned with a particular epistemological, philosophical or theoretical approach. This method encourages thick description and pays attention to the complex layers of meaning and understanding (38). Constant comparative content analysis was then utilized and involved an iterative process of moving backwards and forwards between transcripts, coding and analyzing passages. This method uses systemic coding and categorizing to determine patterns of words and phrases used (39, 40). By comparing, the researcher is able to better understand inductively, by categorizing and coding categories and connecting them (41). To further enhance rigor of the comparative content analysis the researchers (KF, SM) followed a 4-step framework by Whittemore et al. (42), by first paying attention to the voices of participants; second reflecting on how believable the results are; third critically appraising all decisions made during the research process; and lastly researchers (KF, SM) demonstrated ongoing reflection and self-criticality.

A document analysis approach was used as an independent source of evidence as a complement to the interviews. The answers to the key questions provided context resulting in a better understanding of the health system response (43). Triangulation of results obtained from semi-structured interviews and organizational report documents solidified our findings. Triangulation is the combination of methodologies in the same study looking at the same phenomenon which helps to increase validity, decrease researcher bias, and provide additional perspectives of the phenomenon being studied (44). For both the document analysis and comparative content analysis, researchers (KF, CP) were in contact with organizational leads to ensure data interpretation was accurate and appropriate.

Our final step for analysis was sharing our findings back to Indigenous communities for interpretation in a sharing circle format in both an urban and rural context. Through the sharing circle discussion, participants confirmed the findings and no major changes in analysis were identified. Community presentations and a report summarizing the research findings were also shared with the Community Advisory Committee. With the final results, a knowledge sharing and exchange forum with other disaster scholars who conducted research in the region following the wildfire was hosted in RMWB by the study PI (SM). The forum brought together community members, service providers, and representatives from the municipality to hear the findings from this study and others. This forum allowed for dialogue between researchers, community members, service providers and policy, and decision makers which informed local emergency preparedness and policy change at the community level.

### Ethical Considerations

The study was reviewed for its adherence to ethical guidelines and approved by a Research Ethics Board at [University of Alberta REB Protocol # Pro00070845]. All service provider participants were informed and fully consented to participate in the study. Participants were assured that they could withdraw from the study up until data was analyzed without any consequences. All personal details were kept confidential and secure.

## Results

Service providers described their experience of providing support during and following the wildfire disaster. Three superordinate themes emerged from analyses of the interview data: (1) service provision in response to Indigenous mental health concerns, (2) gaps in Indigenous health services post-wildfire; and (3) adopting a health equity lens in emergency management. Findings from the document analysis supported these themes and added further context by highlighting contextual considerations for health system responsiveness including community engagement and ownership, culturally appropriate healthcare access, sustainable and consistent funding for health programming, and recognition of increased vulnerability to public health emergencies.

### Service Provision in Response to Indigenous Mental Health Concerns

In response to the negative impacts from the wildfire on mental health among Indigenous residents and communities, mental health and wellness services and supports were provided in the region's First Nation and Métis communities. Local service providers described an increase in the availability of mental health services which included counseling, psychosocial interventions, and outreach support. A community health worker described how pre-wildfire there was a lack of mental health services and supports for Indigenous communities, and only in times of a crisis are “*services pushed at you when it's chaos, and then when everything is settled, the services are all depleted” (Participant 9)*. While temporary services and supports were delivered to address the immediate risks and impacts to mental health, participants described an “*Indigenous mental health service gap”* that existed in the region before the wildfire. Service providers expressed concerns about how temporary mental health services and/or supports in community underscores the inequity of mental health service provision for Indigenous peoples. This was confirmed in the *Rebuilding Resilient Indigenous Communities* report which highlighted the challenges of intercultural communication and collaboration as well as the context of colonial legacies and their impact on how Indigenous peoples were affected by the wildfire. The report overall described how the evacuation and response lead to inconsistent and inequitable service delivery partly due to the inadequate representation of Indigenous leadership in the Regional Emergency Operations Center and was further hindered by lack of coordination between local Indigenous governments and municipal and provincial governments. This was further eluded to by service providers when commenting on the jurisdictional complexity of the region when it comes to providing services for Indigenous residents.

Additionally, participants stated that the provision of mental health and wellness services and/or supports to rural and remote Indigenous communities were generally sporadic and inconsistently delivered pre-wildfire and that this was significantly heightened post-wildfire. For instance, mental health therapists and cultural liaison workers were hired by the provincial health authority on limited-term contracts, to visit communities in rural and remote areas of the region one-to-two days per week. A health director from a remote community explains the challenges faced by community residents when accessing mental health and wellness services that are not consistently available in the community:

…*we didn't have a consistent counsellor every day of the week or you know, they didn't have regular appointments. She did have a few and then they kind of tapered off after, um, after she was recommending them to come into town. She [counsellor] still comes and we would like her to come more often than not, but it has to do with their funding and having somebody, and resources, to be able to come out. I think the mental health system and team is spread pretty thin (Participant 24)*.

Many local service delivery organizations also adapted their services and collaborated with other agencies in order to respond to the immediate needs of Indigenous residents and communities. Organizations such as the provincial health authority quickly mobilized to develop resources such as mental health and wellness pamphlets, online tools for coping with stress, delivering emergency preparedness kits to communities, and supporting recovery and healing by hosting community gathering events. However, in KPMG's (Klynveld Peat Marwick Goerdeler) *Post-Incident Assessment Report* (p. 99) they state “*… while a re-entry booklet was prepared and provided to residents as they resettled in the Region, it contained limited information regarding access to other resources, supports and services that may have been necessary to help residents with their longer term resiliency*” (45). Concerns relating to Indigenous peoples' mental health in the region were expressed by all study participants. A frontline worker conveys the increased need for mental health services following the wildfire:

*[…] We are being more available for them [clients]. We are more aware of mental health now, whereas it was something that was on our radar pre-fire, but now we find that a lot of people are struggling and need the support now […] (Participant 19)*.

The need for mental health supports was also echoed in the document review. A massive jump in referrals (20,000 in 51 days compared to typical 1,200 per year) was received by local addictions and mental health staff between May 10 and June 30 (~2 months post-wildfire) was identified in the *Post-Incident Assessment Report* (KPMG, 2017). In the same report, RMWB residents themselves identified a strong need for mental health supports and noted they had faced difficulties accessing supports. However, this increase in referrals was reported to include all population groups in the region.

Furthermore, participants described the ways in which their organization quickly adapted their services during wildfire recovery to maintain critical access to services and respond to worsening health and mental health concerns among Indigenous residents. One participant described how their organization was receptive to change and quick to adapt so that their clients have continued access to needed services and supports: “*That's what this team is built all on right there is adaptability. Meeting the person where they're at, or community where they're at, and knowing and respecting the boundaries”* (Participant 6). For service providers to meet the needs of Indigenous residents and communities and provide culturally relevant care, sustainable and long-term funding and equitable access to resources was highlighted as necessary as described in the *Rebuilding Resilient Indigenous Communities* report. As previously mentioned, participants noted gaps in mental health service delivery for Indigenous people during and following the wildfire as a barrier to service provision.

Health directors and community leaders played an important role to ensure delivery of “*culturally responsive mental health services and supports” (Participant 24)*. All Indigenous providers and some non-Indigenous providers in the study had described advocating for mental health services to include traditional aspects of healing with a trauma-focused lens long before the wildfire happened, and even more so following the disaster. A director of an Indigenous service organization shared how his staff turned to traditional and cultural healing practices to respond to trauma and stress within communities:

*Specifically, for mental health we had to get creative with some of our service providers with the trauma counselling, relying on the holistic approach in taking more of a cultural, spiritual route for mental health and healing (Participant 8)*.

The documents included in our review described 18 programs for the prevention and promotion of mental health (Supplementary Table 3) that were promoted in the region. To improve access to mental health services, the provincial health authority opened a free walk-in clinic in urban Fort McMurray from 2016–March 2018 and then from the Canadian Red Cross from April 2018 to March 2021 which also included a mental health therapist to travel to the rural Indigenous communities once a week.

Local providers also helped residents to adjust with their current circumstances by preparing them for future emergencies. Some participants explained that the stress Indigenous residents experienced was in part due to them being unprepared when the wildfire happened effecting their ability to cope. One community worker described how she supported Indigenous elders to prepare for future evacuations:

*They have their medication lists now. They have those tucked away, in a little grab bag, like a book bag that we have prepared. In the book bag it has a couple of pairs of panties, it has a pair of underwear, some socks, um, a clean shirt and their medication. A couple of them have little candies in there, just in case they need to grab that really quick, then they'll have little things that they can kind of nibble on […] (Participant 1)*.

This participant went on to describe that while it is impossible to be fully prepared when a traumatic event occurs, it offers people a sense of safety and control enabling people to cope better under stress.

Moreover, community connection can have positive effects on mental health and well-being (46). In the *CARE Wood Buffalo* report, mental health was ranked the most common barrier faced by residents, and family and socializing were rated highest for what improved residents' well-being post-wildfire. Many local service organizations worked together to mobilize and organize several community gatherings such as block parties. A community coordinator described how the event brought together all residents (Indigenous and non-Indigenous) and how the events were also an opportunity to distribute mental health resources (e.g., pamphlets or booklets on mental health and violence prevention services and supports available in the region and/or how to promote positivity and self-care):

*We did lots of public community events and free events with no stigma attached. Just come out, have fun. We had lots of mental health resources available for anyone that came in, but we didn't force anyone to take anything. So, we just opened conversation with the attendees (Participant 2)*.

The document review also provided detailed information about funding from charitable, municipal and provincial sources to local organizations in Fort McMurray to organize monthly block parties in urban Fort McMurray and rural Indigenous communities outside of the city.

### Gaps in Indigenous Health-Related Services Post-wildfire

In describing the gaps in services for Indigenous residents and communities across the region, several participants emphasized the need to improve Indigenous people's access to high-quality and culturally relevant health and wellness services. Many of the services and programs that were delivered following the wildfire were limited in scope and resources. The provincial health system is also very regimented on funding travel for service providers. The main programing mentioned in the document review was the Canadian Red Cross caseworkers available at the Nistawoyou Friendship Centre as the only Indigenous-specific Welcome Centre for re-entry. Given the short-term funding allocated to specific services and programs this offered little opportunity to foster provider-client relationships. A frontline rural coordinator described her experience by stating,

*It's temporary services … once you've finally engaged the people into a program, then it's gone the next day, you know. It's not enough personnel to cover how many people need to be seen here. Then at the same time, it's not even enough time to engage the people to get to know them, because they only pop in once a week. And how do you build a relationship with a community in one day in a week. I've been here for eight years and I'm still doing it… Their [mental health therapist] workday starts at 8:30am, they get here by 10:30am, they leave by 2:00pm and we have 400 people out here that want to see this one person (Participant 11)*.

As a result of the temporary nature of many new service provision positions that were created to respond to the increasing demand for mental health and wellness services, provider turnover in the community was high, leading to inconsistent and sporadic service delivery. A social worker explains, “*turnover in Fort McMurray is a revolving door. Once you make a relationship with someone [a service provider], then six months later someone else, so you have to start all over again” (Participant 25)*. A local community coordinator further emphasized the need for consistent services to support ongoing healing from trauma: “*from the healing and recovery perspective as well as for individuals, whether that's trauma from their past, growing up, environmental impacts, social impacts, the consistency on the level of supports that are coming in is very important” (Participant 8)*. Frontline providers and staff explained that recovery from traumatic experiences such as the wildfire takes time and thus, sustainable and long-term planning for service provision is required. It was noted that immediately following the wildfire there was an influx of temporary services to support early recovery; however, service providers felt that services and supports for long-term recovery were overlooked. A community health worker in one rural Indigenous community shared:

*It is kind of like victim services. They're there at the forefront and then they're not there when everything actually is calm, the shock is done, um, that's when the people really need to come (Participant 9)*.

Some participants also described how the temporary services were not equipped to address pre-existing health, mental health, and social concerns in the community, which were heightened after the wildfire. A local community worker shared,

*We are losing resources, but I could speak on behalf of the health centre because I was here at the time. There was no programming here. It was a lack of programming in the health centre in general, not much direction. So, our NNADAP [National Native Alcohol and Drug Abuse Program] programme was lacking. There was no pre- and post-natal. We had no public health. We had no home care services. We had no services. All we have here is a building planted on the reserves (Participant 11)*.

These findings suggest that new services and programs delivered to Indigenous residents and communities were not adequately resourced to respond to the determinants of Indigenous peoples' mental health and well-being. Also, participants spoke about the lack of cultural appropriateness among service providers who were deployed to the region. Because of the short-term funding for new services and programs, and especially when providers are out in the community once a week, there was mistrust among community members in providers outside the community. Additionally, lack of trust in governments and organizations from community members resulting from a history of being under-serviced and under-resourced and the added fall out from gaps in responses post-wildfire, was echoed in the document review:

*The lack of trust coloured perceptions of the RMWB's response to the wildfire, particularly in rural areas: ‘I can say that it was a long standing issue with respect to being under-serviced by the municipality in the rural areas. And the fall out of the fire was added to a long list of shortcomings that the rural community has felt that they weren't receiving since amalgamation. So certainly that the whole fire response and the Fort McMurray focus kind of played into that* *(**47**)*.

With outside service providers spending 1 day a week in the rural communities, participants were critical of the quality of the services provided and noted several gaps. For instance, participants spoke about limited engagement and relationship-building with Indigenous clients and local health directors and staff in the communities, and how community members were unaware of new services and/or programs offered in their community. An Indigenous community health worker shared,

*There wasn't a lot of relationships built from the services providers coming from Fort McMurray, I worked long time with these people [community members] and trust doesn't come easily to Indigenous people, it would have been helpful to see a familiar face (Participant 6)*.

Participants also shared their concerns about new services that were perceived as not being culturally appropriate or attuned to the social and cultural realities of Indigenous peoples. Another community health worker stated:

*So we want to have a lot of cultural components that's attached to the serviced offered, because we find that's important now, post wildfire believe it. And I'm sure it is always important, it's always important to me on a personal level. But I think post wildfire, everybody has just kind of come back to the roots, because there's nothing like a great big wildfire to bring you back to humility, right (Participant 19)*.

A youth/cultural coordinator from a rural community stressed the importance of community engagement as a critical first step in tailoring health services to the needs of Indigenous communities. She explains that a lack of engagement with the community had reflected on the number of community members who utilized the services: “*…they don't do community engagement. Then they wonder why their numbers are low. Then they feel that there's not even a need to be out here.” (Participant 17)*. This quote reflects the need for Indigenous voices to be included in the design and evaluations of programing. This was supported in the *Rebuilding Resilient Indigenous Communities in the RMWB* report which focused on the effects of the wildfire on Indigenous peoples and highlighted the importance of inclusion of Indigenous voices for recovery following a public health emergency.

### Adopting a Health Equity Lens in Post-disaster Recovery

Adopting and applying a health equity lens in post-disaster recovery planning was described by participants as essential to reducing disproportionate impacts on Indigenous residents and communities and creating a more sustainable and equitable approach to responding to health-related impacts following the disaster. Health equity refers to the commitment to work toward eliminating disparities in health and to strive for the highest possible health standard for all people (48). Reducing Indigenous health inequities includes promoting the delivery of culturally-safe and equitable care (49). The impact of colonial legacies and the need for reconciliation was emphasized in the *Rebuilding Resilient Indigenous Communities in the RMWB* report (47), and the need to improve culturally appropriate healthcare access was a major focus from the *Athabasca Tribal Council (ATC)* report (50). An important piece to improve cultural appropriateness is by ensuring community supports are Indigenous led and evaluated. Unlike the other reports included in the document analysis, the *Rebuilding* report (35) and the *ATC* report (36) were both led by Indigenous organizations. The *Rebuilding* report discussed a research project on the impacts of the wildfire on Indigenous peoples, and the *ATC* report shared the results of a conference held to identify Indigenous communities' health and wellness needs and gaps in service post-wildfire.

Many participants described the gaps in Indigenous health services in relation to physical accessibility of services (e.g., staff turnover, inconsistent and sporadic service delivery, the number of service providers in the community) and the funding received to organize and deliver services and/or supports. Several other participants went on to discuss the critical role service providers can play to improve access to services by ensuring services are culturally safe, appropriate, and relevant to community needs. These participants emphasized that cultural awareness and an understanding of the effects of colonialism and intergenerational trauma are critical to providing culturally safe and culturally appropriate care for Indigenous peoples across the region. Several participants also recommended that cultural training be mandated for all non-Indigenous service providers outside the community. As stated by a director of an Indigenous-led organization:

*I think it starts with understanding the historical events with first going back to intergenerational trauma, the impacts of, not just residential but also the industry and the impacts that that took on the rural communities as well. Having that knowledge beforehand will allow a person to have a little bit more compassion, understanding and patience is the biggest thing. Because it takes a lot for anybody to go in to a service provider to ask for help (Participant 23)*.

Alternatively, a few participants advocated for dedicated funding from government to train and hire local Indigenous service providers. Concern regarding lack of Indigenous support workers and a preference for Indigenous-led support programs was expressed by Indigenous focus group participants in the *Rebuilding Resilient Indigenous Communities in the RMWB* report (47). On the other hand, non-Indigenous service providers also highlighted the limitations they experienced because of resource and funding inequities which consequently left them feeling incapable of fully meeting the expectations of Indigenous residents, families, and communities. While gaps in service provision were described by participants, feedback from service providers on how to improve service delivery in a post-disaster context were captured.

Some participants shared how power dynamics between service providers and clients/patients can pose barriers to accessing care from a service provider. A community coordinator explained that service providers need to remove their professional cloak to connect with their client/patient and work to establish trust with them. She goes on to state, “*you are wearing a professional hat, but you're really being a friend and that's what they need instead of saying, here, go figure it out, this is the number, call them [referring to a detox centre]” (Participant 1)*. A frontline worker reflected on how she meaningfully engaged with community leaders, residents and other local health workers to support “*knowledge and understanding of what's happening on types of services and supports provided” (Participant 18)*.

The importance of improving culturally appropriate emergency response and healthcare access was discussed in three documents (47, 51, 52). Indigenous recovery post-wildfire was hindered by a lack of Indigenous support workers and Indigenous re-entry points–initially, most of the Welcome Centres were located in schools, without regard for how this could be a barrier for residential school survivors (47). A need for culturally safe support was identified, and the Nistawoyou Friendship Centre became the site of a re-entry point a few days after the initial re-entry centres were opened (52). Some participants stressed how important it is for outside service providers to understand the local context before visiting the community. They pointed out that a “*one size fits all approach”* to service delivery is not feasible as there is much diversity across Indigenous communities and between urban and rural Indigenous populations. This was described by a social worker:

*If we gave it [mental health resource pamphlets] to the community [rural reserve], the community [rural reserve] wouldn't know what to do with it. And so, that was something that I voiced, I helped develop a community-based plan that kind of took all of the elements of what they were saying, but made it kind of, you know, more plain English, more, um, user friendly I should say. They [helping organization] were making a plan for the community to use to recover, but they were doing it in such a high level, it wasn't community based. Community members would just look at it and become probably more anxious, you know, like, I don't know what this means, how am I supposed to recover if I don't know what it means? (Participant 7)*

Thus, being attuned to the social and cultural realities of Indigenous clients/patients was believed to strengthen and promote positive provider-client/patient relationships.

Some participants highlighted the ways in which their organization adapted their procedures and policies to address equity and change how Indigenous clients are supported in their healing and recovery. One provider explained this well:

*For medical appointments, we do referral services, so if there's something that we can't help in. So, we have a client, let's say they'd called us or reached out to us either in urban or [rural reserve] community, and there, like, you know what, I really want to go detox or I really need to see mental health [support]. Then we don't just say, well, here's the number and call them, we'll say let's call them. So, we'll support them though that whole process and not just say, here, do it on your own, because sometimes when you're at that point you can't do it on your own. It was hard enough to ask for help (Participant 22)*.

Breaking down systemic barriers and advocating on behalf of clients was discussed in much detail by some participants. Despite systemic challenges, one mental health staff person described her commitment to advocate and goes on to say “*if I see things that were unjust or if, um, I see that there's possibilities, um, or things that are not being done that could be done, um, you know, I'm going to rally with them to get done” (Participant 7)*.

Community leaders and frontline staff also described how Indigenous models of health and healing can be used to guide the delivery and implementation of community-led health services and programs in the community. As one public health nurse shared:

*We include the medicine wheel and we just try to make it culturally appropriate with some of the stuff we gave, like little handouts, like for aboriginal youth or HIV. So, we try to make it culturally appropriate… We do have a working group with elders, women's groups, and more so turned out to be, in general a woman's support group where we worked with traditional medicines and cultural activities (Participant 9)*.

Furthermore, individual and family-centered approaches to care were also advocated for to align services and programs with the needs of Indigenous residents, families and communities. A service team lead recounted how her team went out of their way to support clients:

*I know we've done so much for so many people and it will be really, if people would ask, like, if they asked for something or something in particular, we'd go on the hunt and find [it]. Like, I know one family, she was really into, um, her beading and making moccasins and stuff like that. So, we went to Halfords and had her whole box of supplies waiting for when she moved in so she could have some basic tools to start doing what she loved again. And that's how we got back to bringing it right down to people. We talked to people, what do you need and what do your family need? Not so much looking on the greater scale sometimes, but focusing in and zoning in on that one family, how do we meet your needs (Participant 5)?*

## Discussion

This study described how mainstream health systems (health service-delivery organization across sectors) responded to the health and mental health needs of Indigenous residents following a wildfire disaster. The conceptual framework by Mirzoev and Kane (11) and Valentine et al. (21) for health systems responsiveness guided our data interpretation (11). The Mirzoev & Kane (11) framework “positions the experience of interaction between people and health system as the centerpiece and recognizes the determinants of responsiveness both from the health system (e.g., actors, processes) and the people (e.g., initial expectations) sides” (11). The service providers shared their experiences as actors following interactions within the health system as well as their perception of expectations and experiences of their Indigenous clients.

We applied the concept of health system responsiveness, originally developed by the WHO, and adaptations of the concept and frameworks. Our study findings present opportunities to explore health system responsiveness in relation to the realities of Indigenous peoples' within the context of a wildfire disaster. Our findings build on, and advance, core frameworks on health system responsiveness to propose new elements for a framework for health system responsiveness aligned with the realities and experiences of care for Indigenous peoples in the context of a public health emergency.

One of the elements of the Mirzoev and Kane (11) framework for assessing health system responsiveness is on the characteristics of health services (e.g., availability, accessibility, and quality). Findings from service provider interviews and the document review demonstrated that the delivery of health-related services in Indigenous communities following the wildfire were shaped by pre-existing health and mental health service gaps in the region, historical funding inequities toward Indigenous health, temporary funded service provider positions during wildfire recovery, and inconsistent delivery of programs and services throughout the wildfire; which ultimately meant that the needs and expectations of care for Indigenous residents and communities were not adequately met. Prior to the wildfire, Indigenous peoples in Canada faced precarious access to health services and had more unmet health needs compared to the non-Indigenous population (53, 54). Public health emergencies disrupt availability of services, cause damage to physical infrastructure, and engender psychological or mental distress which create increased need for mental health services and supports for individuals and families.

Service providers advocated for sustainable and long-term supports during a crisis and highlighted the missed opportunity to foster provider-client relationships with temporary services and inconsistent presence of service providers in the community. This is compounded by a distrust among Indigenous peoples with the health system because of past and current experiences with racism, discrimination and oppression in the Canadian healthcare system, leading to the perpetuation of health inequities (55). As trust is another identified element in the health system responsiveness framework by Mirzoev and Kane (11), future research is needed to examine the relationship between the sustainability of services and programs offered in communities and uptake or use of those services and programs by Indigenous people.

Cultural safety and cultural responsiveness are also an important determinant that shapes Indigenous users' experience across the health system. Contextual considerations such as historical traumas and current systemic racism greatly impact Indigenous people's interaction with health service-delivery organizations. Findings from the document analysis and service provider interviews offered suggestions for improving Indigenous residents' experiences with a health system following a public health emergency. This included providing cultural sensitivity training and education as a part of reconciliation to address colonial legacy and historical traumas. Minnican and O'Toole (56) categorized characteristics of culturally responsive communication for service providers to be reflexive, flexible, self-aware, respectful of diverse cultures, transparent, and non-judgemental (56). Noted in our interviews, service providers touched on similar characteristics of Minnican and O'Tooles (56) findings and also suggested education among providers to bring awareness about the local Indigenous context and cultural diversity (for instance, awareness of Dene, Cree, and Métis culture of the RMWB region).

Service providers highlighted the role of community engagement to build relationships, improve understanding of local contexts for service providers and foster community connectedness, which in turn supported emotional well-being. Moreover, connection to the land, culture, and spirituality must be considered when responding to mental health needs of Indigenous populations (4). As an effort to normalize mental health disturbances experienced by the residents following the wildfire, Alberta Health Services (AHS) launched a campaign called “Recovery Takes Time” and emphasized that recovery looks different for everyone (57). This highlights the importance of tailoring resources and supports for diverse population groups and across intersections of gender, sex, culture, ethnicity, race, and Indigeneity. Furthermore, service providers in our study were critical of the limited mental health resources and supports available to Indigenous communities, and especially for rural communities. Funding and resources allocated to mental health service delivery for Indigenous populations during a public health emergency should account for how services and supports are organized and delivered. Our findings emphasized delivering services in a timely, culturally relevant and culturally safe manner, in order to best support Indigenous communities during and following a public health emergency.

All interviewed participants spoke about mental health support as a crucial aspect of recovery and the document review revealed positions that were created for mental health and psychosocial support (47, 58, 59). Several documents reviewed had reported on increase utilization of mental health services and supports, includes both Indigenous and non-Indigenous population groups in their reporting and mis-represents the experience of Indigenous people. In contrast, the interviews commented on lower utilization of support services and barriers to access for communities. In addition, while organizations documented programs and service availability in Indigenous communities, service providers discussed the inadequate amount of time allotted to programing and that temporary support did not meet the needs of communities.

Moreover, as some service providers commented on the jurisdictional complexity of the RMWB it is important that we understand the health systems' response to the health and mental health needs of Indigenous peoples following the wildfire within the context of the dual funding system (federal–provincial) for Indigenous health. The jurisdictional relationship between the Federal and provincial governments has generated tension regarding who is responsible for funding health, resulting in confusion, set-backs, resentment, and failure to address healthcare in Indigenous communities, not only related to funding for health initiatives but also with components that impact the determinants of health. Whereas, there is a fiduciary obligation of the federal government around Indigenous health in Canada, there has been a clear lack of federal leadership in emergency management for Indigenous communities. For instance, Indigenous Services Canada (ISC) delegates programs to the Alberta Emergency Management Agency (AEMA) but First Nations reserves remain outside of provincial jurisdiction and there is very little coordination support and guidance provided by ISC. As a result, there was inadequate coordination between the RMWB, the Province of Alberta, and First Nations during the 2016 wildfire, which enhances jurisdictional territoriality and impedes cooperation and coordination of services and supports to communities (47). Indigenous organizations and local health service centers were reported as being underfunded and understaffed, impacting their ability to be prepared for public health emergencies (47).

There have been frequent crises that disproportionately affect Indigenous communities in Alberta over the past decade or more. These include the 2009 H1N1 epidemic, the 2013 floods in southern Alberta, the 2016 wildfires in northern Alberta, the opioid epidemic, and the recent COVID-19 pandemic. In all of these situations, delays in determining responsibility for the response were experienced, despite fiduciary obligations of the federal government to Indigenous peoples as well as jurisdiction over reserve lands, but with provincial governments tasked with health services supports delivery. The wildfire as well as the COVID-19 pandemic highlighted the problems that fragmented or “siloed” health and social care systems face in adapting to crises that require an urgent and collaborative response (60–62). With each of these emergencies or crises, health systems, and governments have been tested to demonstrate how care and supports can be organized and delivered rapidly, yet the relationships between decision-makers, providers, and community leaders have had to be reformed each time. The wildfire and COVID-19 pandemic in particular have demonstrated most prominently that community engagement, community leadership, and knowledge of Indigenous communities is an essential foundation for public health during moments of crisis.

The framework for Indigenous Disaster and Emergency planning developed by Montesanti and colleagues (63) from previous research on a major flood in First Nation communities in southern Alberta, provides a promising resource to guide future disaster response and recovery in Indigenous communities by addressing the social determinants of health and supporting community-led response to disaster recovery. This framework highlights several key characteristics discussed in this manuscript such as a holistic understanding of health and wellness, community-led emergency plans, and recognition that colonialism and racism still exist and are to be discouraged. Thus, our research findings presented in this paper enrich our understanding of the key characteristics outlined in the framework and can be used to inform local emergency responses at the community level. The overall structure of disaster and emergency management programs and policies has emerged from the dominant political system and has been overlaid on Indigenous communities. The results of this system exclude the voices of Indigenous peoples from public health emergency response, and ultimately result in continuing colonization through dominant disaster and emergency management programs and policies. Our document review underscored the exclusion of Indigenous communities in the planning of evacuation and response. By increasing awareness of the health and social inequalities in risk management, it will be possible to engage in risk reduction planning with communities and promote community-led and culturally safe responses to public health emergencies. Climate change is projected to continue to drive increased risks over the coming decades, risks that will be compounded by non-climatic factors such as social, economic, cultural, political, and institutional inequities. It is important to understand how disaster response and emergency planning measures can play a role in reducing harm and promoting healing instead of perpetuating vulnerabilities and health and social inequities.

Health system responsiveness could be improved by encouraging community control over what services are provided (64). Self-determination not only leads to more appropriate services but also contributes to reconciliation as a tool to reduce the oppressive legacy of colonization and historical traumas (65). Further, similar to prior disaster response in Indigenous communities, jurisdictional and governance challenges were noted and lack of communication between leadership in communities was observed to influence the health systems' response (4). Prior research stresses the importance of coordination and collaboration between government and organizations that are supporting mental health and recovery (66). Inter-agency cooperation and collaboration were briefly discussed in the interviews and document analysis but was not identified as a common theme. Where collaboration of service delivery was discussed, Indigenous focused interagency collaboration was not mentioned.

It is important to note that community members perceptions and experiences were shared through the lens of the service providers. This is a possible limitation for this work as we relied on the input of service providers to understand health system responsiveness in the context of Mirzoev and Kane's conceptual framework. However, in our other work led by authors of this paper (KF, SM, TA, TM, LA) (4), community members did speak to the health systems response following the wildfire disaster. Our research findings provide insights into the development of an adapted health system responsiveness framework which acknowledges Indigenous peoples experience with the health system during a public health emergency. Based on our findings the following domains of responsiveness are critical to advocate for: (1) access to cultural safe and culturally responsive care; (2) trust between service providers and clients; (3) respect for Indigenous culture and knowledge; (4) inclusion of Indigenous values in the design and delivery health services; and (5) attention to equity. Further development of these domains of responsiveness needs to be explored and validated by Indigenous peoples and experts. Additionally, determinants of responsiveness for Indigenous peoples may be shaped by allocation of resources, health system organization, and historical and institutional factors.

## Conclusion

This research examined how health systems responded to the immediate and long-term health and mental health needs among Indigenous residents and communities following the Horse River wildfire in northern Alberta, Canada. Interviews with health service providers and a review of available organization and government reports provided key information on the provision of health and mental health services following the wildfire, gaps in service delivery, socio-political factors that shaped delivery and access to health-related services, and suggestions for strengthening responsive health systems for Indigenous health. The health system responsiveness concept was used to guide data interpretation and the application of proposed frameworks on health system responsiveness, to understand Indigenous peoples' interaction and experience with health services provided following the wildfire. Our findings demonstrated that the needs and expectations of care for Indigenous residents and communities following the wildfire were not adequately met. For instance, funding and resources for Indigenous health services was limited and in general not culturally safe or relevant. However, many service organizations did demonstrate how they worked with what they had and collaborated with other agencies to provide Indigenous peoples in the region with access to needed health and mental health services and adapted and implemented new delivery approaches to promote culturally-responsive care. A main service adaptation in response to the crisis, included an increased availability of mental health services such as counseling, psychosocial interventions, and outreach support. Adopting and applying a health equity lens in post-disaster recovery planning was highlighted as essential to reducing the disproportionate impacts on Indigenous residents and communities and creating a more sustainable and equitable approach to responding to health-related impacts following a public health emergency. Furthermore, attention to the roots of disaster and the colonial process of disaster and emergency management programs and policies can help Indigenous communities to heal and recover from a public health emergency.

## Data Availability Statement

The original contributions presented in the study are included in the article/Supplementary Materials, further inquiries can be directed to the corresponding author.

## Ethics Statement

The study was reviewed for its adherence to ethical guidelines and approved by a Research Ethics Board at the University of Alberta (Pro00070845). All service provider participants were informed and fully consented to participate in the study. Participants were assured that they could withdraw from the study up until data was analyzed without any consequences. All personal details were kept confidential and secure. The participants provided their written informed consent to participate in this study.

## Author Contributions

SM led the funding acquisition for the study and is the Nominated Principal Investigator for the funding grant. SM and KF led the writing of the manuscript. SM and TW designed the interview guide in partnership with the Community Advisory Committee. SM and a community research assistant conducted interviews. SM, TW, KF, and TA developed the coding framework for analysis of the interviews. KF and TA completed initial data analysis, and KF and SM refined the key themes. KF and CP conducted the document review and extraction (led by CP). CP contributed to the manuscript writing and formatting. JS and LA confirmed results and contributed to manuscript writing. All authors reviewed the manuscript and provided edits and feedback.

## Funding

The research presented in this manuscript was funded by the Canadian Institutes for Health Research (CIHR)–Health Effects Alberta Wildfires Operating Grant (151026).

## Conflict of Interest

The authors declare that the research was conducted in the absence of any commercial or financial relationships that could be construed as a potential conflict of interest.

## Publisher's Note

All claims expressed in this article are solely those of the authors and do not necessarily represent those of their affiliated organizations, or those of the publisher, the editors and the reviewers. Any product that may be evaluated in this article, or claim that may be made by its manufacturer, is not guaranteed or endorsed by the publisher.
