# Supplementary material for: Health Systems Responsiveness in Addressing Indigenous Residents' Health and Mental Health Needs Following the 2016 Horse River Wildfire in Northern Alberta, Canada: Perspectives From Health Service Providers
Source: Front Public Health. 2021 Dec 2;9:723613. doi: 10.3389/fpubh.2021.723613 (PMC8704385; doi:10.3389/fpubh.2021.723613)
Supplement: Supplementary file 1 [file Image_1.pdf]

Interview Guide

We're Interested to learn a bit about yourself, your role within your organization, and knowledge gained through the events of the wildfire as we look to learn about the impact and recovery as it relates to the health and wellness of Indigenous groups and people of RMWB.

Before we begin with the interview, can I ask if you identify as Indigenous?

1. What is your current role within your department or organization? Were you in this role during the 2016 wildfire?

- If so, what have you experienced in your role through events of the fire?
- What was it like in the first months of returning to and serving in the community after the evacuation?

If not, what has your experience been since commencing your current role?

2. Can you describe what health and wellness, and/or social support services your department or organization provides within the Regional Municipality of Wood Buffalo?

[Probe: How are these services or programs delivered?]

3. Does your department or organization provide direct services to Indigenous residents of RMWB? If yes, both urban and rural residents? For rural, which communities?

4. Did events of the fire have an affect to your department or organization? [Probe: did the fire pose any constraints (financial, resource, or other) to your department or organization?

5. Have there been noticeable changes to your organisation with relation to the services and supports provided for the Indigenous populations of Wood Buffalo post-wildfire? [Probe: shortage of staff resulting from not returning back after the evacuation]. Has there been specific set backs (give examples) to your department since the fire? If so, can you explain what this has been like, how your managing them, as well as what you feel is required to help further at this time?

6. From your professional experience, how do you perceive the impacts from the fire on the health and mental health of Indigenous residents? Can you comment on any specific health or mental health issues that resulted from the fire, evacuation, relocation, and resettlement? Do the impacts differ for urban Indigenous evacuees, on-reserve First Nations and Métis communities? If you don't feel you can provide an adequate response to this question, is there an employee/staff person in your department/organization that who might have this knowledge?

7. Have events of the fire affected and or changed the delivery of services you offer to Indigenous people and communities in the region? If yes, can you explain? [Probe: has there been special attention to the needs of Indigenous residents and the provision of services to address the needs of Indigenous residents impacted by the fire?]

8. Are you aware of any other changes to the provision of health and wellness and/or social care support services in RMWB (e.g., services that might no longer be provided or additional services or supports offered to Indigenous residents to support their recovery and health post-wildfire)?

I would like to ask some questions specifically to the mental health impacts and mental health services provided to Indigenous peoples and communities post-wildfire [These are questions for providers involved in mental health service delivery]

9. How are Indigenous residents coping after the wildfire? Can you describe any mental health concerns experienced by Indigenous residents as a result of the fire?

10. Can you describe the organization and provision of mental health services in the RMWB? (e.g., AHS run programs/facilities, community/non-profit, Indigenous-led mental health supports). How do these services differ for on-reserve and in urban Fort McMurray? Do Indigenous residents use mental health

11. How many mental health referrals has your department or organization made or received? What is the referral process in your organization for patients to mental health services or supports?

12. Can you describe the type(s) of mental health services and programs that are offered in the RMWB? (e.g., counselling, outreach, treatment for post-traumatic stress, substance use/addictions, etc.).

13. Do you perceive any gaps in mental health supports or programs offered to Indigenous residents?

Questions related to culturally appropriate care:

[Preamble] People who have experienced trauma are at risk of being re- traumatized in every social service and health care setting. We know that intergenerational trauma as well as direct, indirect and vicarious experiences of trauma (such as the wildfire) have impacted the health and well-being of Indigenous peoples.

14. What practices and processes are applied within your department or organization to support trauma-informed care with Indigenous children, youth and families/communities who have experienced traumatic events like the 2016 wildfire?

***Questions related to recommendations for service delivery*:**

15. How to improve services for Indigenous communities and people at this time? Or how could services have been improved at time of the fire?

16. Through and following the fire, what has been working, what isn't going so well, and or 'what can be done better' how can this be improved?

17. What do you believe to be most important as it relates to recovery for Indigenous peoples and communities in RMWB?

Questions related to Data:

18. Has your organisation been involved in gathering data/ information as it relates to the programs and services you offer to Indigenous people and communities? If so, can you explain more details about this and the process by which it was gathered?

Questions relating to the health and mental health impacts of service providers (including front-line staff) from providing care and support to Indigenous evacuees and those impacted from the fire.

19. How were you personally impacted by the 2016 wildfire?

20. Do you feel comfortable sharing what your experience has been with providing care and support for Indigenous residents in the aftermath of the wildfire? Have you experienced any personal difficulty with caring for residents who have been traumatized or negatively impacted by the wildfire?

21. What measures are you taking to protect your own mental health?
