# Supplementary material for: Health Systems Responsiveness in Addressing Indigenous Residents' Health and Mental Health Needs Following the 2016 Horse River Wildfire in Northern Alberta, Canada: Perspectives From Health Service Providers
Source: Front Public Health. 2021 Dec 2;9:723613. doi: 10.3389/fpubh.2021.723613 (PMC8704385; doi:10.3389/fpubh.2021.723613)
Supplement: Supplementary file 2 [file Table_2.pdf]

Table 2: Document Extraction Results

Questions	AB Fire One Year Report Canadian Red Cross	RMWB 2016 Recovery Plan	KPMG Post-Incident Assessment Report for AB Emergency Management Agency	RMWB Lessons Learned and Recommen-dations Report	Wildfire Funding Partnerships Red Cross (webpage)	Wildfire Partnership Decision Table Terms of Reference (TOR) Red Cross	ATC Health & Wellness Report	Rebuilding Resilient Indigenous Communities in RMWB Report	CARE Wood Buffalo Report
Where did the funding come from? How much funding was provided to organizations for communities to respond to the wildfire?	$323 million raised by public and governments. $244 million spent at time of publication.	$615 million from Federal Disaster Financial Assistance Arrangements and provincial Disaster Recovery Program. $319 million from CRC. $3.6 billion from Insurance Bureau of Canada. "Significant" money from "individuals, businesses,	~$319 million from CRC.	$30.3 million from Government of Alberta in donation matching. Of that: $14.3m to local charitable, non-governmental and Indigenous organizations; $15m for small business owners; $1m for health research and initiatives. $3.853m from CRC and $10.5m from Government of Alberta	N/A	$299m raised CRC, $50m of which allocated to Community Partnerships Program. United Way of Fort McMurray also received ~$3m.	Funding supplied by Canadian Red Cross, dollar amount not stated.	Funding supplied by Canadian Red Cross, dollar amount not stated.	Funding supplied by Canadian Red Cross, dollar amount not stated.

		and other NGOs”		for FireSmart.					
Was any of this funding for Indigenous specific initiatives/activities/programs/service?	Yes; Nistawoyou Friendship Centre.	Unclear.	Unclear. Recommended continuing to fund FireSmart programming for interested Indigenous communities.	Yes. Creation of Indigenous Art Piece project, and other unspecified grants for Indigenous organizations.	Yes. Funded initiatives with various Indigenous organizations and governments. Also provided financial assistance for Indigenous People’s Day in RMWB in 2018.	N/A	Yes, all was used for the ATC Health & Wellness Project.	Yes, all of it was used for focus groups, interviews, a survey, and a review of secondary data.	No, however encouraged to include Indigenous communities in events and outreach
What was the decision-making process for how funding was distributed to different service providers/organizations/stakeholders?	Unclear.	Unclear.	Unclear.	Unclear.	N/A.	Applications and decisions for funding were reviewed by the Community Partnerships Table, made up of representatives from Canadian Red Cross	N/A	N/A	Application submitted by AHS in collaboration with YMCA, Waypoints, CMHA, Athabasca Tribal Council, Centre of Hope and other local service providers. Decision to

						(1, co-chair); United Way of Fort McMurray (1, co-chair); Indigenous leadership (1); RMWB Administration (1); Government of Alberta (1); Industry Recovery Working Group (1); Community Wellness and Resiliency Committee (1) and the Social Recovery Task Force (2).			fund made by Canadian Red Cross

What was the duration of this funding?	Unclear. Document was produced one year after the fire and some projects were receiving continued funding.	Unclear.	Unclear.	Unclear.	Unclear.	N/A	Unclear, appeared to have lasted from 2018-2019.	Research lasted for nearly two years.	2017-2019
Was there a sustainability plan implemented? If so, what does this look like?	Unclear. $2.8 million of funds raised were allocated towards future disaster planning and preparation.	The recovery plan extends for 3 years. Key action areas are: people, environment, economy, rebuild, and mitigate.	A variety of recommendations are made but is is unclear whether a specific sustainability plan was created.	Sustainability of FireSmart was discussed and progress of recovery plan from 2016 document was also discussed.	N/A	N/A	Yes; 5-year plan was created and will be partnering with communities and local leaders and champions to receive input and to access sustainable funding.	The intent of the project was to develop recommendations to ensure that "reconstruction and future-risk-mitigation plans contribute to enhancing the resilience and adaptive capacity of Indigenous communities" (p.7)	No

How many positions were created to respond to the wildfire? Were any of these positions to address Indigenous specific initiatives/activities/programs/services?	Unclear.	Unclear.	AEMA added 6 Regional Field Officers to its Field Operations Unit and 2 Field Officers for First Nations communities.	2 positions created by partnership between RMWB, McMurray Métis, and ATC to help First Nations and Métis communities in Wood Buffalo with recovery. Also, two engagement strategists representing the communities were embedded within the Stakeholder Engagement Team of the Recovery Task Force.	N/A	12 volunteer positions for Community Partnerships Table.	12 volunteers at Health & Wellness Conference. Unclear if any others; most work done by ATC staff.	Unclear.	N/A

What was the mandate from organizations or stakeholders for communities to address the health and wellness and/or mental health of RMWB residents following the wildfire?	A mandate is not discussed but the number of individuals provided with mental health and support programming and events is given.	Their mandate included needing to care for one another's mental and physical health.	A mandate is not discussed but the number of individuals seeking psychosocial supports after evacuation is given.	Unclear. There was a Psycho-Social Framework for recovery in Wood Buffalo supported by a Community Wellbeing and Resiliency Committee.	Unclear.	N/A	Intent was to understand how to improve health & wellness supports to be culturally appropriate for ATC's First Nation communities.	Unclear.	A healthy community brought together through in-person connection and empathetic understanding - where every member feels supported in their own way and no one is left behind.
Does this report include findings from wildfire specific programing? Any health-related outcomes?	No.	No, but desired outcomes are listed.	The outcome of the Information Centres for community re-entry and what they provided was discussed.	The opening of the Indigenous Welcome Centre was an outcome.	N/A	N/A	The findings from the Health & Wellness Conference were grouped into 4 themes which were then connected to goals within the 5-year plan created.	Health effects were reported in interviews, focus groups, and the survey. Effects included respiratory illness, stress, anxiety, substance use, and other addictions. Negatives impacts on self-reported health were more significant within the communities	Through CARE WB there were 5,614 homes visited in the community of Fort McMurray. Out of these homes, a total of 1,514 (27%) residents were available to connect with a volunteer. There were a total of 70 follow up referrals that were provided by CARE Wood Buffalo

								that were evacuated.	volunteers.A total of 9 community engagement events were hosted within the RMWB. That included rural Indigenous communities

What were the lessons learned?	Unclear.	Unclear. It is outlined as part of the plan to conduct an analysis to gather lessons learned.	Identified lesson that stakeholders needed to be better engaged, earlier on, and that Indigenous stakeholders needed to be engaged in different ways than other stakeholders. Other lessons learned were not specific to Indigenous communities.	Identified need to prioritize disaster preparedness and community resilience. Suggested the needs of all RMWB communities, including Indigenous ones, should be included in the Municipal Emergency Management Plan or that they should have community-specific supplements. Open lines of communication between RMWB and Indigenous communities when creating emergency management plans and during emergencies	N/A	N/A	Four themes were identified from the conference. 1: Youth leadership – more programming and development is needed, and should be sustainable and culturally appropriate. 2: Land-based education and language revitalization – increase programming for all ages 3: Culturally appropriate health care accessibility – access to culturally appropriate health care related to all aspects of the medicine wheel. Also identified	Identified a lack of preparedness from RMWB, inadequate support and communication breakdowns from RMWB and AEMA, lack of coordination between different levels of government, a lack of clarity of roles and responsibilities. Also found a lack of safe and culturally appropriate evacuation sites.	(1) The door-to-door campaign and community engagement events were effective in improving the wellbeing of the residents in the Regional Municipality of Wood Buffalo through sharing of resources, referrals, and conveying messages of compassion and understanding. (2) There is direct intervention and therapeutic value in providing psychosocial support and listening to residents share their story. (3) Residents were able to share their stories,

				was also emphasized.			addictions as a growing issue. 4: Proactive First Nation communities . Increasing engagement and events and planning to be proactive rather than reactive.		express concerns, and engage with volunteers from the safety of their own homes and in a non-clinical setting.<br>(4) Relationships among community partners and services providers strengthened and collaboration increased(5) The CARE Wood Buffalo project required flexibility, in that reaching residents in the rural communities was more efficient through community engagement events instead of door-to-door.<br>(6) The RMWB is a

									very multicultural and linguistically diverse region, therefore, language barriers made it challenging for volunteers to engage with several residents.<br>(7) required many volunteers in order to be successful, with high burnout rates after the fire it was difficult for volunteers to continue to commit to the project.
