# Supplementary material for: Health Systems Responsiveness in Addressing Indigenous Residents' Health and Mental Health Needs Following the 2016 Horse River Wildfire in Northern Alberta, Canada: Perspectives From Health Service Providers
Source: Front Public Health. 2021 Dec 2;9:723613. doi: 10.3389/fpubh.2021.723613 (PMC8704385; doi:10.3389/fpubh.2021.723613)
Supplement: Supplementary file 3 [file Table_3.pdf]

Table 3: Mental health programs (services, supports, interventions, etc.) that were offered or promoted during the wildfire for residents in the region.

**Sponsoring Organization**	**Program Offered (n=18)**	**Predated Wildfire?**
Canadian Red Cross (CRC)	Set up welcome/information/reception centres during re-entry. Initially, there were seven throughout the region; a few days later, one at Nistawoyou Friendship Centre was launched for Indigenous community members after cultural inappropriateness of other locations was identified. AHS mental health teams were present at centres, and CRC caseworkers provided mental health support in the months following the evacuation at Nistawoyou Friendship Centre.	No
First Nations and Inuit Health Branch (FNIHB), Government of Canada	FNIHB "deployed a support team to assist First Nations peoples in their evacuation and re-entry" per RMWB PSS report by policy wise	No
Indigenous communities, with funding by CRC	Hosted Indigenous recovery events e.g. Healing Gathering for the Land and Water, National Aboriginal Day celebration in Fort McMurray.	Unclear
Alberta Health Services & others	Mental health teams were available for firefighters and first responders as well as the public; they were present in Fort McMurray from the start and also at welcome centres during re-entry. There was an Indigenous Traveling Team by AHS for Fort McMurray and area Indigenous communities.	No
Various organizations, with funding by CRC	Multiple re-entry/welcome events were held by a variety of organizations and funded by CRC.	No
Government of Alberta	School-based supports, including First Nation, Métis, and Inuit mobile outreach success coaches, were provided.	No
Government of Alberta	Planned funding for health initiatives and research activities to promote mental health for residents affected by the wildfire.	No
CARE Wood Buffalo	Hosted monthly community block parties in urban and rural communities and conducted a door to door campaign in urban Fort McMurray with the goal of providing awareness of resources and supports available in the community.	No
Association canadienne-francaise de l'Alberta and Centre d'accueil et	Needs assessment of Francophone community in Fort McMurray, French-language mental health services and programming in the region.	No

d'établissement du Nord de l'Alberta, with funding by CRC		
Canadian Mental Health Association, Alberta North East Region, with funding by CRC	Increased capacity of existing Pets and Wellness (PAWs for People) pet therapy program.	Yes
Canadian Mental Health Association, Alberta Division, with funding by CRC	Family Peer Support Network for parents and caregivers of children and youth experiencing mental health concerns.	Unclear
CRC	Financial support for mental health supports for students and staff across four school districts in Fort McMurray Public Schools.	No
Northern Lights Regional Health Foundation, with funding by CRC	Toolkits and age-appropriate learning materials to support the psychosocial and mental wellbeing of preschool-aged children.	Unclear
Canadian Mental Health Association North East Region, with funding by CRC	Community engagement and capacity building to support those at risk of suicide, and community-led events to provide overall mental wellness and coping tools.	Unclear
Keyano College, with funding by CRC	Development of campus-wide mental health and wellness programming.	No
Regional Municipality of Wood Buffalo (RMWB) Recovery Task Force	Psychosocial support was offered to 2,200 teachers.	No
RMWB Recovery Task Force	Hosted one-year anniversary event for residents.	No
RMWB Recovery Task Force	Hosted Health and Wellness Workshops for general residents and also specifically for teachers and students.	No
